# The effect of COVID-19 pandemic on sleep-related problems in adults and elderly citizens: An infodemiology study using relative search volume data

**DOI:** 10.1371/journal.pone.0271059

**Published:** 2022-07-12

**Authors:** Eun Jung Cha, Hong Jun Jeon

**Affiliations:** 1 Department of Psychiatry, Konkuk University Medical Center, Seoul, Korea; 2 Department of Psychiatry, School of Medicine, Konkuk University, Seoul, Korea; UNITED KINGDOM

## Abstract

COVID-19 has had a substantial national impact in South Korea, causing negative psychological responses including sleep-related problems. Literature indicates sleep problems among the general population have been reported to be as high as around 35.7% during the first 8 months of COVID-19. Therefore, the aim of this study was to investigate the impact of COVID-19 pandemic on sleep problems among the general population using relative search volume (RSV) data, and whether there are any differences by age and time periods spanning before and during the pandemic. RSV data was collected from the most commonly used search engine in South Korea, NAVER. Search terms were grouped into 4 categories: insomnia, other sleep disorders, sleeping pills, and sleeping pill side effects. Time points were divided into 4 periods, each 7 months long: 7 months before COVID-19 (T0), first confirmed COVID-19 case to 7 months after (T1), 7 to 14 months (T2), and 14 to 21 months (T3). A 2x4 factorial Analysis of Variance was conducted to investigate main effects and interactions between age and time periods. Main effects and interaction effects of age and time periods were significant for all search term groups. For all search terms, both age groups showed dramatic increase from T0 to T1. In age group 60 or above, RSV continued to increase for other sleep disorders and sleeping pill. Insomnia and sleeping pill side effects showed decreasing trend at T3. In general, sudden increase in RSV after occurrence of COVID-19 followed by slow decline were observed. However, for age group 60 or above, RSV values of other sleep disorders and sleeping pills continued to increase, suggesting slower recovery of psychological impact with increasing age. Overall, the results underscore the importance of implementing preventive measures for monitoring sleep problems during the pandemic, especially in the elderly.

## Introduction

Ever since the novel COVID-19 first emerged, it spread rapidly, quickly becoming a pandemic. COVID-19 is a highly infectious respiratory disease that can be fatal, in particular to the elderly and people with pre-existing medical conditions. In South Korea, the first confirmed case of COVID-19 occurred on January 20^th^, 2020 [1]. While initial speed of infection was slow, a superspreading event that occurred during March 2020 dramatically increased the number of confirmed cases [2]. Combined with the abrupt increase in confirmed cases both nationally and globally, there have been detrimental economic and financial impact, such as financial loss due to strict social distancing restrictions. These negative effects have caused negative emotional reactions, leading to overall decline in mental health of the general population. Thus, studying the psychological impact of the pandemic has become an important mission to better the lives of people affected by the pandemic. This has been evidenced by the growing interest to study the impact of COVID-19 on mental health.

Various types of psychological responses to the COVID-19 pandemic have been observed worldwide. Commonly studied responses indicated elevated depression, anxiety, stress, and loneliness levels [3, 4]. A somewhat less frequently studied response is sleep problems. Sleep problems are commonly reported in those with mood disturbances, such as anxiety and depression [5, 6]. It is necessary to address sleep-related response, as sleep problems may result in serious mental and physical health consequences, including increased emotional distress and mood disorders, decreased quality of life and memory performance, and increased risk of cardiovascular disease [7]. In elderly, sleep problems can lead to decreased physical function, cognitive impairment, and even mortality [8]. Therefore, sleep is an important aspect to examine when evaluating risks for decreased mental and physical health.

Researchers have advocated for certain actions to be taken in awareness of the negative responses, such as providing psychological interventions for affected communities, and addressing and monitoring mental health in addition to physical health at healthcare facilities [9, 10]. As such, investigating sleep problems during the time of COVID-19 is vital for increasing the understanding of sleep problems and thus develop appropriate interventions. Related meta-analyses reported the global pooled prevalence rate of sleep problems during COVID-19 to be as high as 35.7% and sleep disturbances to be 40.5% [11, 12]. Because it has been two years since COVID-19 has been declared as pandemic, sleep problems in the general population may have experienced changes with time such as increased prevalence in other sleep disorders.

When investigating public health responses to pandemic, Internet search data is highly useful, especially in the current society where Internet search is automatic when faced with a problem [13]. Internet search data can be analyzed in the form of relative search Volume (RSV). RSV data indicates how much a certain search has been made over a certain period of time. NAVER, the most commonly used search engine in South Korea, offers NAVER Trends service that provides RSV data, which can be computed according to gender and age range. As South Korea ranks among the highest Internet penetration rate, majority of the general population employ Internet searches, including the elderly. A recent study demonstrated the utility of RSV data by employing NAVER search volumes to successfully build predictive models of new cases and deaths in South Korea [14]. Therefore, this study aimed to investigate the impact of COVID-19 on sleep problems among the general population using RSV data, comparing the changes by time points and age. Based on the available evidence, we hypothesized that, 1) search volumes relating to sleep problems will increase after COVID-19 outbreak, and 2) the pattern of changes in search volumes will differ by age.

## Method

### Data collection

This study was an infodemiological study that analyzed data collected online [15]. RSV data were collected from NAVER. As of October 2020, NAVER occupied 65.13% of total searches in the health/medicine category, ranking as South Korea’s most commonly used search portal, according to data anonymously collected from monthly active users [16]. The search terms were categorized into four groups, each reflecting different aspects of sleep-related problems: insomnia, other sleep disorders, sleeping pills, and sleeping pill side effects. Sleep disorders were divided into insomnia and other sleep disorders, as insomnia is a commonly occurring sleep disorder in response to the stressful pandemic situation [17]. Within each group contained its relating search terms: 1) ‘insomnia’ included ‘insomnia’, ‘sleep quality’, and ‘length of sleep’; 2) ‘other sleep disorders’ included ‘sleep disorders’, ‘restless leg syndrome’, ‘snoring’, ‘sleep apnea’, ‘rapid eye movement (REM) behavior disorder’, ‘sleepwalking’, ‘sleep paralysis’, and ‘hypersomnia’; 3) ‘sleeping pill’ included ‘sleeping pill’ and ‘sleeping aid’; and 4) ‘side effect’ included ‘sleeping pill side effects’, ‘sleeping pill dependency’, ‘sleeping pill withdrawal’, and ‘sleeping pill addiction’.

Due to the nature of RSV data increasing with time, it was crucial to control each time period to be approximately equal to each other. Therefore, we divided the period after COVID-19 outbreak into three sections, each 7 months long. To provide a baseline measure, we have also included data from 7 months period before the COVID-19 outbreak. The time period before and after COVID-19 were divided into four periods, each 7 months long. The first occurrence of COVID-19 patient in South Korea was on 2020 January 20^th^. Therefore, period before COVID-19, named T0, spanned 2019 June 20^th^ to 2020 January 19^th^. Consequently, T1 spanned 2020 January 20^th^ to 2020 August 19^th^, T2 spanned 2020 August 20^th^ to 2021 March 19^th^, and T3 spanned 2021 March 20^th^ to 2021 October 19^th^. The time periods contained minimum of 424 days and maximum of 429 days. As for age, separate RSV data for each age group was exported: 60 and above, and 19 to 59. The entire procedure was reviewed and approved by the Institutional Review Board (KUMC 2021-11-030).

### Statistical analysis

The data were first tested for normality. The data were revealed to be skewed, and were thus log-transformed. The quantile-quantile plots and histograms of the transformed data resembled that of normal distributions, and were evaluated as eligible for parametric analyses.

The data were analyzed using 2x4 factorial Analysis of Variance (ANOVA), with between-subjects variable set as age group (60 and above, 19 to 59) and within-subjects variable as time points (T0 through to T3). Analyses were performed for each search term groups, thus resulting in four factorial ANOVAs. Effect sizes were calculated using η^2^. Significant interaction effects were elucidated via post-hoc pairwise comparisons that employed paired *t*-tests. Effect sizes of the *t*-tests were calculated using Cohen’s *d*. All statistical significance was tested at alpha level of 0.05. Statistical analyses were performed using IBM SPSS Statistics for Windows, Version 27.0 (Armonk, NY: IBM Corp.).

## Results

**Fig 1:**
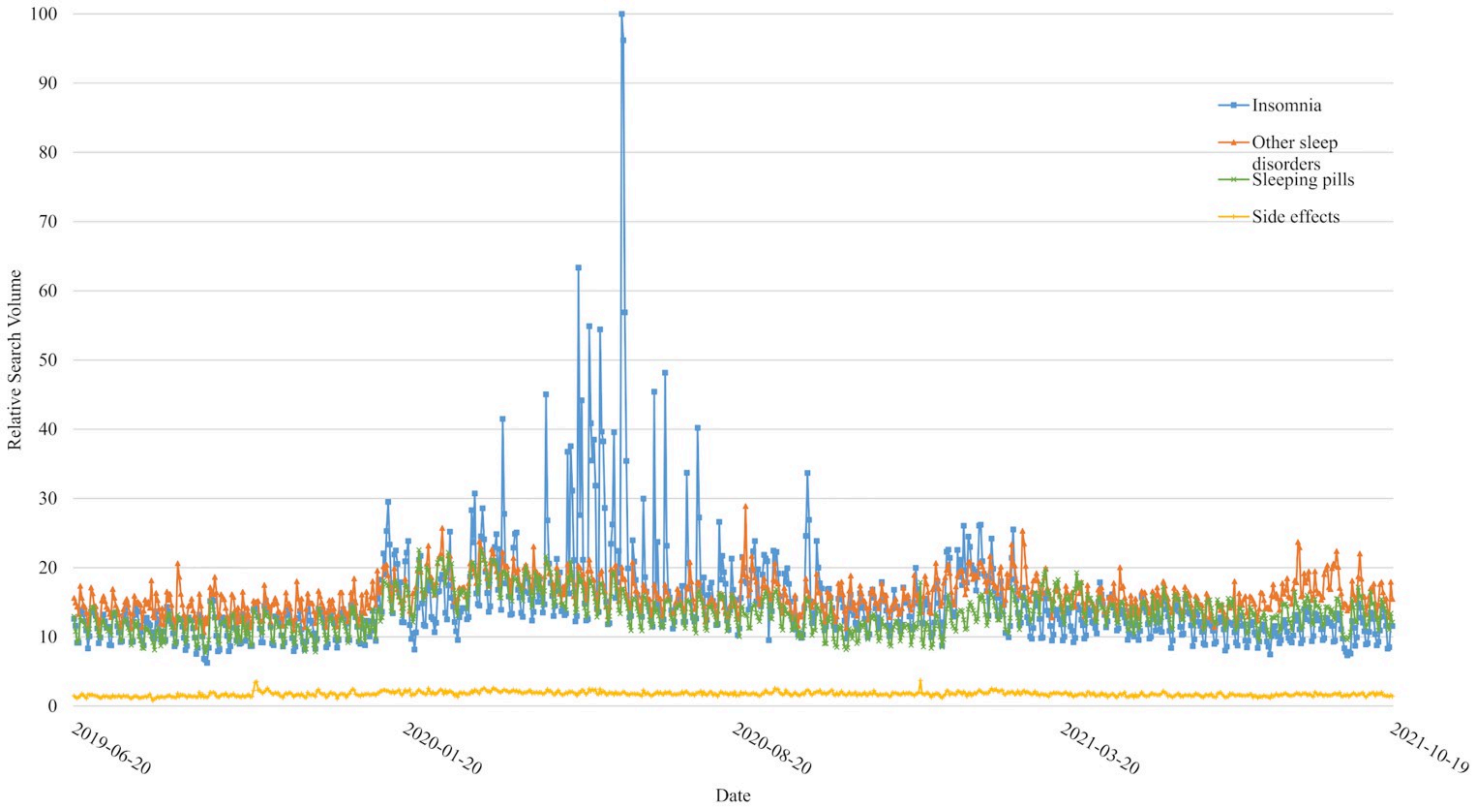
RSV trends of searches related to insomnia, other sleep disorders, sleeping pills, and its side effects from 2019 June 20th to 2021 October 19th.

Means and standard deviations of RSV data for each search term categories are described in Table 1, arranged by time and age group. Fig 1 illustrates daily search terms over the time period analyzed for this study.

**Table 1:** Means and standard deviations of RSV data for each search term category, arranged according to age group and time point.

Variable	Age Group	Time Point	Mean (SD^a^)
Insomnia	19 to 59	0	11.95 (3.14)
		1	20.01 (12.08)
		2	15.25 (4.18)
		3	11.49 (2.03)
	60 or above	0	15.69 (5.79)
		1	26.77 (11.30)
		2	26.57 (8.76)
		3	19.79 (5.27)
Other Sleep Disorders	19 to 59	0	14.40 (1.97)
		1	17.30 (2.51)
		2	16.36 (2.48)
		3	15.39 (2.03)
	60 or above	0	21.49 (5.73)
		1	27.97 (8.57)
		2	30.26 (10.74)
		3	31.21 (8.14)
Sleeping Pill	19 to 59	0	11.36 (2.03)
		1	15.83 (2.78)
		2	12.42 (2.31)
		3	13.12 (1.79)
	60 or above	0	20.22 (8.23)
		1	36.15 (11.19)
		2	32.10 (11.25)
		3	37.42 (8.29)
Side Effects	19 to 59	0	1.55 (0.36)
		1	1.84 (0.25)
		2	1.72 (0.26)
		3	1.49 (0.19)
	60 or above	0	5.06 (2.40)
		1	7.25 (2.95)
		2	9.24 (3.40)
		3	8.27 (2.74)

^a^SD indicates standard deviation.

The period between each labelled date indicates T0, T1, T2, and T3, in chronological order. First COVID-19 case was observed on 2020 January 20^**th**^. Superspreading event occurred on 2020 March.

### Insomnia

For insomnia, main effects of age and time were both significant (F(1, 1698) = 733.48, η^2^ = 0.30, *P* < .001; F(3, 1698) = 228.11, η^2^ = 0.57, *P* < .001, respectively). Age by time interaction was also significant (F(3, 1698) = 23.82, η^2^ = 0.04, *P* < .001).

Further comparison tests revealed that for age group 19 to 59, searches for insomnia significantly increased from T0 to T1 (t_(425)_ = 13.20, *P* < .001, *d* = 0.15), and significantly decreased from T1 to T2 (t_(423)_ = 5.83, *P* < .001, *d* = 0.15), and T2 to T3 (t_(424)_ = 12.21, *P* < .001, *d* = 0.10). Significant difference between T0 were also observed at T2 (t_(424)_ = –9.90, *P* < .001, *d* = 0.11), such that more searches were made at T2. At T3, there was no significant difference between the number of searches compared to T0 (t_(426)_ = 1.21, *P*>.99, *d* = 0.09).

**Fig 2:**
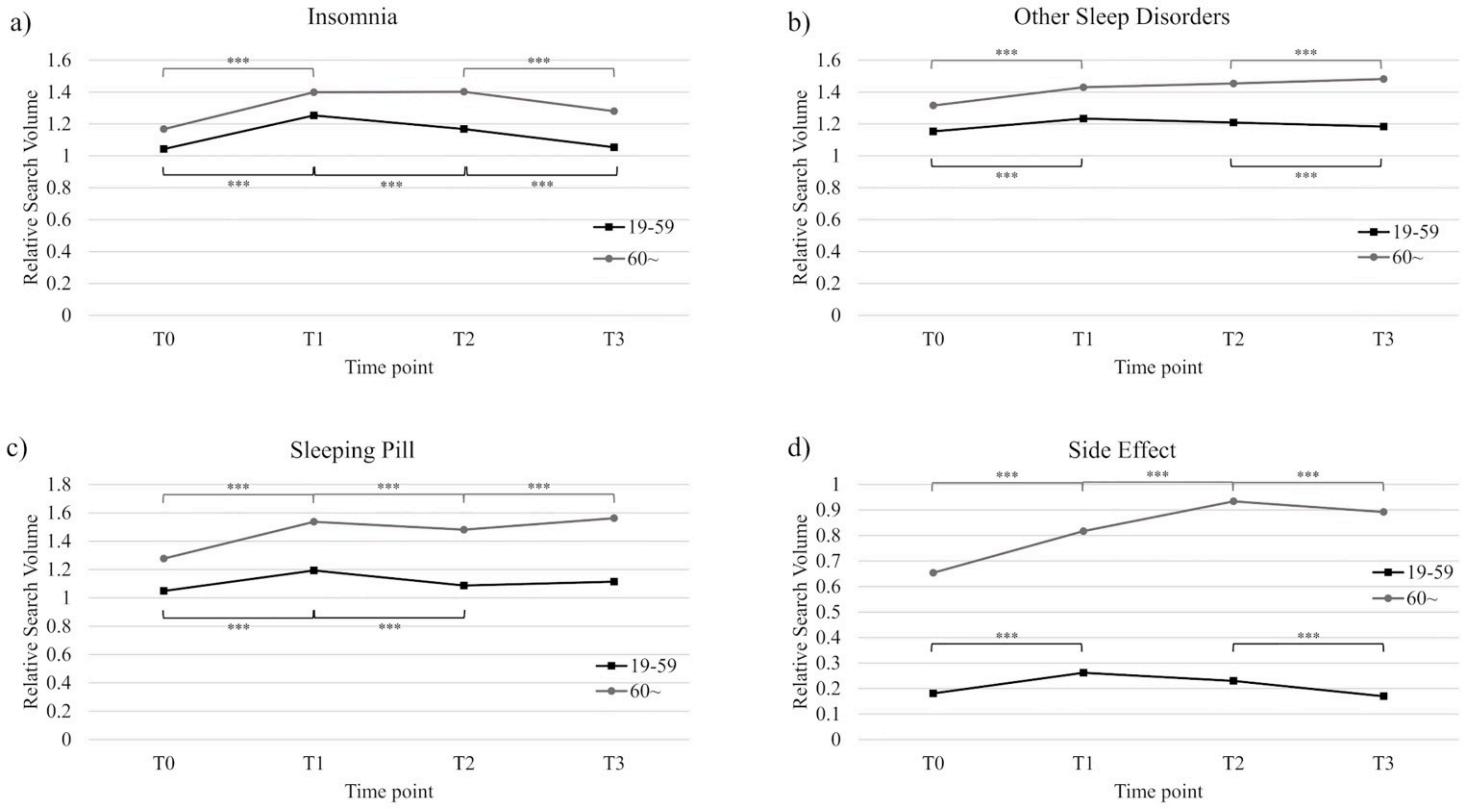
Log-transformed RSV data by age groups at time points T0 to T3. (a) Insomnia. (b) Other sleep disorders. (c) Sleeping pills. (d) Side effects.

For age group 60 or above, searches for insomnia significantly increased from T0 to T1 (t_(425)_ = –15.40, *P* < .001, *d* = 0.16). Unlike age group 19 to 59, the increased search was maintained until T2, where it significantly decreased to T3 (t_(424)_ = 9.59, *P* < .001, *d* = 0.13). Significant differences between T0 were observed at T2 (t_(424)_ = –16.26, *P* < .001, *d* = 0.15) and T3 (t_(426)_ = –8.27, *P* < .001, *d* = 0.14), such that more searches were made at both time points compared to T0. The results are summarized in Fig 2a.

### Other sleep disorders

For other sleeping disorders, main effects of age and time were both significant (F(1, 1698) = 2245.53, η^2^ = 0.57, *P* < .001; F(3, 1698) = 103.47, η^2^ = 0.16, *P* < .001, respectively). Age by time interaction was also significant (F(3, 1698) = 38.82, η^2^ = 0.06, *P* < .001).

Further comparison tests in age group 19 to 59 revealed that searches for other sleep disorders significantly increased from T0 to T1 (t_(425)_ = –13.43, *P* < .001, *d* = 0.06), and decreased from T2 to T3 (t_(424)_ = 4.38, *P* = .047, *d* = 0.06). Comparisons between searches at T0 revealed significant differences with T2 (t_(424)_ = –9.20, *P* < .001¸*d* = 0.06) and T3 (t_(426)_ = –5.33, *P* = .012, *d* = 0.06), such that searches at both time points were greater than those of T0.

For age group 60 or above, similar to age 19 to 59, searches significantly increased from T0 to T1 (t_(425)_ = –9.83, *P* < .001, *d* = 0.12). However, searches also increased from T2 to T3 (t_(424)_ = –2.16, *P* = .03, *d* = 0.13), unlike age 19 to 59. Searches were significantly greater at T2 (t_(424)_ = –10.36, *P* < .001, *d* = 0.14) and at T3 (t_(426)_ = –15.34, *P* < .001, *d* = 0.11) compared to T0. The results are summarized in Fig 2b.

### Sleeping pills

Main effects of age and time were both significant (F(1, 1698) = 4434.91, η^2^ = 0.72, *P* < .001); F(3, 1698) = 285.30, η^2^ = 0.34, *P* < .001). Interaction for age and time was also significant (F(3, 1698) = 78.42), η^2^ = 0.12, *P* < .001), warranting for further comparison tests.

Further comparison tests revealed that for age group 19 to 59, searches relating to sleeping pills significantly increased from T0 to T1 (t_(425)_ = –19.35, *P* = .001, *d* = 0.08), and significantly decreased from T1 to T2 (t_(423)_ = 13.79, *P* < .001, *d* = 0.08). However, searches at both T2 and T3 were still greater than T0 (t_(424)_ = –4.98, *P* = .002, *d* = 0.08; t_(426)_ = –9.82, *P* < .001, *d* = 0.07, respectively).

For age group 60 or above, searches significantly increased from T0 to T1 (t_(425)_ = –18.42, *P* < .001, *d* = 0.15), decreased from T1 to T2 (t_(423)_ = 4.01, *P* < .001, *d* = 0.14), and increased yet again from T2 to T3 (t_(424)_ = –6.65, *P* < .001, *d* = 0.13). Similar to age 19 to 69, searches at T2 and T3 were all significantly greater than T0 (t_(424)_ = –13.91, *P* < .001, *d* = 0.15; t_(426)_ = –22.91, *P* < .001, *d* = 0.13, respectively). The results are summarized in Fig 2c.

### Side effects

For searches relating side effects of sleeping pills, main effects of age and time were both significant (F(1, 1698) = 7755.25, η^2^ = 0.82, *P* < .001; F(3, 1698) = 101.73, η^2^ = 0.15, *P* < .001, respectively). Interaction of age and time was also significant (F(3, 1698) = 73.67, η^2^ = 0.11, *P* < .001).

Further comparison tests revealed that for age group 19 to 59, searches significantly increased from T0 to T1 (t_(425)_ = –10.76, *P* < .001, *d* = 0.08) and decreased from T2 to T3 (t_(424)_ = 10.64, *P* < .001, *d* = 0.06). Compared to T0, T2 revealed significantly greater amount of searches (t_(424)_ = –6.44, *P* = .003, *d* = 0.08).

In age group 60 or above, searches significantly increased from T0 to T1 (t_(425)_ = –7.84, *P* < .001, *d* = 0.22), T1 to T2 (t_(423)_ = –6.15, *P* < .001, *d* = 0.20), and decreased from T2 to T3 (t_(424)_ = 2.63, *P* = .02, *d* = 0.17). For all time points later than T0, searches were significantly greater (t_(424)_ = –14.77, *P* < .001, *d* = 0.20; t_(426)_ = –13.12, *P* < .001, *d* = 0.19 for T2 and T3, respectively). The results are summarized in Fig 2d.

Table 2 summarizes the results comparing T0 to time points succeeding the onset of COVID-19.

**Table 2:** Difference of RSV data between post-COVID-19 and pre-COVID-19 for each variable.

Variable	Age Group	Compared Time Point	Difference^a^	*P*	Cohen’s *d*
Insomnia	19 to 59	T1	–0.19	< .001	0.15
		T2	–0.10	< .001	0.11
		T3	0.01	>.99	0.09
	60 or above	T1	–0.23	< .001	0.16
		T2	–0.24	< .001	0.15
		T3	–0.11	< .001	0.14
Other Sleep Disorders	19 to 59	T1	–0.08	< .001	0.06
		T2	–0.06	< .001	0.06
		T3	–0.03	0.01	0.06
	60 or above	T1	–0.11	< .001	0.12
		T2	–0.14	< .001	0.14
		T3	–0.17	< .001	0.11
Sleeping Pills	19 to 59	T1	–0.14	0.001	0.08
		T2	–0.04	0.002	0.08
		T3	–0.07	< .001	0.07
	60 or above	T1	–0.26	< .001	0.15
		T2	–0.20	< .001	0.15
		T3	–0.29	< .001	0.13
Side Effects	19 to 59	T1	–0.08	< .001	0.08
		T2	–0.05	0.003	0.08
		T3	0.01	< .99	0.08
	60 or above	T1	–0.16	< .001	0.22
		T2	–0.28	< .001	0.20
		T3	–0.24	< .001	0.19

^a^Differences are computed by subtracting the value at time point from the value at T0. Negative numbers indicate larger value in the time point stated under “Compared time point”.

## Discussion

This study investigated the changes of general population’s interests in sleep problems using RSV data on insomnia, other sleep disorders, sleeping pills, and its side effects. Comparisons were made by time before COVID-19, onset to 7 months after, 7 to 14 months after, and 14 to 21 months after, and by age, 19 to 59, and 60 or above. Based on related literature, it was hypothesized that searches related to sleep problems will generally increase after the COVID-19 outbreak. The results revealed this hypothesis to be partially true, where a rapid increase just after COVID-19 outbreak was observed. However, searches showed a decreasing trend, with the exception of search terms for sleeping pills and side effects in age 60 or above. Our second hypothesis predicted that the patterns will differ by age group. This was observed for all groups of search terms. For insomnia, age group 19 to 59 returned to baseline at T3, whereas age group 60 or above showed a slower recovery. Age 60 or above also displayed continued increase in searches for other sleep disorders and sleeping pills, as well as a dramatic increase in searches for sleeping pill side effects, unlike the younger age group. The results overall suggested changes in RSV due to age and time point, which can be attributed to different responses to psychological impacts by age, and the onset of the COVID-19 pandemic.

The results generally display dramatic increases in sleep-related searches immediately after the onset of COVID-19 pandemic, indicating increased people experiencing sleep disorders such as insomnia. Pandemics in the past, such as H1N1, have been reported to cause various psychological symptoms such as anxiety [18]. Furthermore, insomnia has also been reported to increase as a result of the ongoing pandemic [19]. This underscores the need to implement policy support that includes psychological preventive measures for sleep disturbances in earlier stages of the pandemic.

Another general pattern observed from the results is the slow recovery towards the baseline level followed by the initial dramatic increase in search rate as the pandemic progresses. This indicates that sleep disorders including insomnia manifest as acute symptoms as a result of psychological stress, which recovers toward baseline as people adjust [20]. One factor that may contribute to this process is resilience, as research has shown it to have a significant inverse relationship with sleep problems such as sleepiness, fatigue, and quality of life [21]. In addition, the results indicate that such return to baseline is not observed for age group 60 or above. In fact, for search categories of other sleep disorders, sleeping pill, and side effects, the results for older group show a continued, yet slower, increasing trend. Such pattern of slower recovery particular to elderly has been observed in previous literature on psychological impact of natural disasters [22, 23]. To our knowledge, this pattern has not previously been observed during pandemic, and is the first to be demonstrated in this study. As sleep quality is a predictive factor of various mental health problems such as depression, anxiety, and even suicidality [5, 6], this result emphasizes the importance of preventive measures for negative impact in the elderly at the beginning of the pandemic.

Interestingly, for sleep disorders other than insomnia, a steady increase in search rates were observed only in age 60 or above. For ages 19 to 59, while the increase in search rates from T0 to T3 was significant, its size is markedly smaller than that of age 60 or above. These results are in line with previous reports of higher prevalence of certain sleep disorders in the elderly, such as REM sleep behavior disorder [24] and disturbances attributed to changes in sleep/wake rhythm [25]. Higher prevalence of sleep disorders in the elderly may be due to insomnia which can be a cause for other sleep disorders, such as sleep paralysis [26] and exacerbation of pre-existing sleep disorders, such as sleep-disordered breathing [27]. Therefore, clinicians treating older populations should carefully assess any occurrence of a new sleep disorder or worsening of pre-existing sleep disorders.

With regards to searches relating to sleeping pills, both age groups showed significant increases during the beginning of the pandemic. This reflects increased need for sleeping pills in response to sleep disturbances such as insomnia. Interestingly, age group 60 or above showed a larger gap of increase as well as continued increase with time, unlike age group 19 to 59. This pattern resembles that of insomnia and other sleep disorders above. Furthermore, in age group 60 or above, a dramatic increase in searches relating to side effects was observed, compared to age group 19 to 59. Sleeping pills such as benzodiazepine drugs or Z-drugs can cause a variety of side effects such as drug dependence. Recently, there have been reports of their long-term effects such as suicide attempts and dementia [28, 29]. As these side effects are typically more serious in elderly population [30], it is concerning that higher search rates on sleeping pill and its side effects are observed in age group 60 or above.

### Limitations

This study has a few limitations that need to be addressed. First, due to the nature of the RSV data, only indirect inferences can be made. It is inappropriate to directly interpret Internet search trends as symptoms actually experienced by the general population, as it would put the study at risk for interpretation bias. Second, Internet searches are not always made in the face of experiencing symptoms. For example, pre-existing sleep conditions may inflate search volumes, regardless of the pandemic context. This may lead to biased data that is greater than the true value. In addition, not all individuals are accustomed to searching their symptoms online. This is especially true for the elderly who may not be familiar or comfortable with employing Internet searches compared to the younger generation. Therefore, the results may not be entirely generalizable to the older population. Third, as this study only investigated results for searches made in a Korean search engine, the results may not be generalizable to other countries speaking languages other than Korean. Finally, this study only retrieved data from a single search engine, which limits the findings to NAVER user pool. Despite the fact that NAVER occupies over half of all health/medicine-related searches made online, it only partially represents online searches made in Korea. If possible, future studies should also consider other search engines that are frequently used, such as Google and Daum that ranks the second and third most used search engines in Korea [16].

## Conclusions

In conclusion, the results generally show a sudden increase in searches following the onset and occurrence of COVID-19 and its superspreading event and a slow decline afterwards. However, this general pattern seems to occur more slowly in the elderly. This difference in pattern by age group can be attributed to slowed recovery from psychological impact with increasing age [22, 23]. In addition, for age group 19 to 59, it was observed that search data did not show significant increase from before COVID-19 for insomnia and side effects related to sleeping pills. As sleep problems have been shown to negatively impact mental health problems, these results support the necessity of placing preventive measures regarding sleep in the elderly during the pandemic. Consistent monitoring is required to sensitively notice and respond to any new onset of sleeping disorders.

## Supporting information

S1 File Raw data used for the analyses. (SAV)

S1 Appendix Sleep-related search terms in Korean. (DOCX)
