# Supplementary material for: The effect of COVID-19 pandemic on sleep-related problems in adults and elderly citizens: An infodemiology study using relative search volume data
Source: PLoS One. 2022 Jul 12;17(7):e0271059. doi: 10.1371/journal.pone.0271059 (PMC9275680; doi:10.1371/journal.pone.0271059)
Supplement: S1 Appendix — (DOCX) [file pone.0271059.s002.docx]

**S2 Appendix. Sleep-related search terms in Korean.**

Insomnia: 불면증, 불면장애

Sleep quality: 수면의 질

Length of sleep: 수면 시간

Sleep disorders: 수면장애

Restless leg syndrome: 하지불안증후군

Snoring: 코골이

Sleep apnea: 수면무호흡

Rapid eye movement behavior disorder: 렘수면행동장애

Sleepwalking: 몽유병

Sleep paralysis: 수면마비

Hypersomnia: 과수면증

Sleeping pill: 수면제

Sleeping aid: 수면유도제

Sleeping pill side effects: 수면제 부작용

Sleeping pill dependency: 수면제 의존

Sleeping pill withdrawal: 수면제 금단

Sleeping pill addiction: 수면제 중독
